# Enzymatic Activity Is Not Required for Phospholipase D Mediated TNF-α Regulation and Myocardial Healing

**DOI:** 10.3389/fphys.2018.01698

**Published:** 2018-11-29

**Authors:** Meike Klier, Simone Gorressen, Marc-Andre Urbahn, David Barbosa, Margriet Ouwens, Jens W. Fischer, Margitta Elvers

**Affiliations:** ^1^Department of Vascular and Endovascular Surgery, Experimental Vascular Medicine, Heinrich-Heine University Medical Center, Düsseldorf, Germany; ^2^Institute for Pharmacology and Clinical Pharmacology, Heinrich-Heine University, Düsseldorf, Germany; ^3^German Diabetes Center, Institute for Clinical Biochemistry and Pathobiochemistry, Düsseldorf, Germany; ^4^German Center for Diabetes Research (DZD), München-Neuherberg, Germany; ^5^Department of Endocrinology, Ghent University Hospital, Ghent, Belgium

**Keywords:** PLD, enzymatic activity, myocardial infarction, ischemia, TNF-α

## Abstract

Phospholipase D1 is a regulator of tumor necrosis factor-α expression and release upon LPS-induced sepsis and following myocardial infarction (MI). Lack of PLD1 leads to a reduced TNF-α mediated inflammatory response and to enhanced infarct size with declined cardiac function 21 days after ischemia reperfusion (I/R) injury. Deficiency of both PLD isoforms PLD1 and PLD2 as well as pharmacological inhibition of the enzymatic activity of PLD with the PLD inhibitor FIPI protected mice from arterial thrombosis and ischemic brain infarction. Here we treated mice with the PLD inhibitor FIPI to analyze if pharmacological inhibition of PLD after myocardial ischemia protects mice from cardiac damage. Inhibition of PLD with FIPI leads to reduced migration of inflammatory cells into the infarct border zone 24 h after experimental MI in mice, providing first evidence for immune cell migration to be dependent on the enzymatic activity of PLD. In contrast to PLD1 deficient mice, TNF-α plasma level was not altered after FIPI treatment of mice. Consequently, infarct size and left ventricular (LV) function were comparable between FIPI-treated and control mice 21 days post MI. Moreover, cell survival 24 h post I/R was not altered upon FIPI treatment. Our results indicate that the enzymatic activity of PLD is not responsible for PLD mediated TNF-α signaling and myocardial healing after I/R injury in mice. Furthermore, reduced TNF-α plasma levels in PLD1 deficient mice might be responsible for increased infarct size and impaired cardiac function 21 days post MI.

## Introduction

Phospholipase D (PLD) belongs to the family of phospholipases, which catalyzes the degradation of phosphatidylcholine into phosphatidic acid (PA) and choline (McDermott et al., 2004) whereby PA itself is a very important second messenger in many cellular processes (Oude Weernink et al., 2007). There are two different isoforms of PLD -PLD1 and PLD2- that have a 50% homologous sequence (Kodaki and Yamashita, 1997) and are expressed ubiquitously in different mammalian cells. Oude Weernink et al. showed that the activity of both isoforms is very different. PLD1 has a low basal activity and is activated by members of the Rho-family (RhoA, Rac1) and protein kinase C (PKC). In contrast, PLD2 exhibits a high basal activity and its activation is only marginally induced by different activators (Oude Weernink et al., 2007).

Platelet activation promotes PLD activity by different platelet activating proteins such as collagen and thrombin (Martinson et al., 1995; Vorland and Holmsen, 2008). PLD1 plays an important role in GPIb-dependent integrin activation and cell adhesion (Elvers et al., 2010). Loss of PLD1 protects mice from arterial thrombosis and ischemic brain infarction while PLD2 deficiency does not alter platelet activation (Thielmann et al., 2012). PLD1 is also involved in platelet-mediated inflammation. It was recently shown that PLD1 is required for platelet–endothelial cell interaction and leukocyte recruitment upon inflammatory processes. PLD1 supports α_IIb_β_3_ integrin activation and firm adhesion of platelets and leukocytes to the inflamed endothelium under high shear conditions (Klier et al., 2017). The impact of PLD1 in inflammatory diseases was also shown in peritonitis (Sethu et al., 2010) and cancer (Gomez-Cambronero, 2010) Moreover, PLD plays a role in myocardial ischemia and reperfusion injury (Schonberger et al., 2014). Loss of PLD1 reduced the elevation of acute phase cytokines including TNF-α and the migration of inflammatory cells into the infarct border zone 24 h post ischemia. Moreover, myofibroblast differentiation and interstitial collagen deposition were altered in *Pld1^-/-^* mice pointing to an important role for PLD1 in TGF-β secretion and α-SMA expression of cardiac fibroblasts. Consequently, infarct size was increased and cardiac function was impaired 28 days after myocardial ischemia in *Pld1^-/-^* mice indicating that PLD1 is crucial for TNF-α mediated inflammation and TGF-β mediated collagen scar formation (Schonberger et al., 2014).

In platelets the loss of both PLD isoforms in *Pld1^-/-^/Pld2^-/-^* mice led to defective integrin activation and agonist-induced P-selectin exposure and protects mice against ferric chloride-induced thrombosis (Thielmann et al., 2012) and stroke (Stegner et al., 2013). Treatment of platelets and mice with the PLD inhibitor FIPI (5-Fluoro-2-Indolyl Deschlorohalopemide) known to inhibit the enzymatic activity of both PLD1 and PLD2 resulted in altered platelet activation and protection against FeCl_3_ injury and ischemic stroke as observed in *Pld1^-/-^/Pld2^-/-^*mice (Stegner et al., 2013). However, the impact of FIPI and the enzymatic activity of PLDs after myocardial infarction (MI) were completely unknown. Thus, we analyzed mice in a model of experimental MI to investigate if pharmacological inhibition of PLD affects the inflammatory response and cardiac remodeling after myocardial I/R injury.

In this study, we were able to show that the enzymatic activity of PLD has no influence on TNF-α signaling and myocardial healing after ischemia and reperfusion (I/R) injury in mice.

## Materials and Methods

### Animal

Animal studies were performed in accordance with the guidelines of the European Parliament for the use of living animals in scientific studies and in accordance with the German law for protection of animals. The protocol was approved by Heinrich-Heine-University Animal Care Committee and by the district government of North-Rhine-Westphalia (LANUV, NRW, AZ 84-02.04.2013.A486).

Gene-targeted mice lacking either PLD1 or PLD2 were described before (Dall’Armi et al., 2010). Both mutant mouse lines were intercrossed to create constitutive *Pld1^-/-^/Pld2^-/-^* mice and the corresponding wild-type littermates were bred from breeder pairs. For the analysis of the impact of PLD enzymatic activity on myocardial ischemia and reperfusion injury, treatment of C57BL/6J mice (Janvier Labs) with the PLD inhibitor FIPI were performed. Experiments were done with male mice aged 10–12 weeks. Mice were anesthetized with Ketamin (100 mg/kg Ketavet^®^, Pfizer, berlin, Germany) and Xylacin (5 mg/kg, Xylazin 2% Bernburg, Medistar, Ascheberg, Germany) by intraperitoneal (i.p.) injection before opening the thorax for removal of the heart or euthanasia was performed by cervical dislocation.

### Myocardial Ischemia and Reperfusion in Mice

For the analysis of MI, 10– to 12-week-old male mice were anesthetized by intraperitoneal injection of a solution with Ketamin (90 mg/kg bodyweight) und Xylacin. Myocardial ischemia was induced by ligation of the left anterior descending artery (LAD) for 60 min. Immediately after ischemia 3 mg/kg bodyweight FIPI/4% DMSO/PBS was injected intraperitoneally. Control mice received 4% DMSO/PBS. A concentration of 3 mg/kg of the PLD inhibitor FIPI was chosen because this dose should provide 20 h of full inhibition (Chen et al., 2012). To this end, FIPI was injected once when the acute phase after myocardial I/R (24 h) was analyzed in mice. Moreover, FIPI injections were performed once daily up to 21 days post myocardial infarction (MI) when we analyzed mice in the chronic phase of myocardial injury.

Twenty four hours after reperfusion, the ischemic area (area of risk) and the infarcted area (infarct size) were determined by staining with TTC/Evans Blue–solution. The ratios of the different areas were quantified digitally by video planimetry.

For the analysis of infarct size in chronic series, infarct size was determined by Gomori’s One step trichrome staining 3 weeks after reperfusion. After progressing successfully anesthesia, hearts were removed, fixed in 4% formalin, embedded in paraffin and cut in serial sections for staining with Gomori’s One Step Trichome solution. The infarct size was expressed as the percentage of the total left ventricular (LV) area.

Echocardiography was performed at different time points using Vevo 2100 ultrasound machine (VisualSonics Inc., Toronto, Canada) and different parameters, e.g., fractional shortening and ejection fraction were determined with corresponding software.

### Isolation of Mouse Embryonic Fibroblasts (MEFs)

For isolation of mouse embryonic fibroblasts (MEFs) embryos from donor mice were prepared at state E12-14. After removing of head and offal, the embryo was comminuted manually and decomposed by 0.05% Trypsin-EDTA and vigorous pipetting three times for 5 min at 37°C. The homogeneous cell suspension was collected in cell culture medium DMEM (Sigma, Darmstadt, Germany) containing 10% fetal calf serum (Sigma, Darmstadt, Germany), 1% Penicillin/Streptomycin (Sigma, Darmstadt, Germany), 1% NEAA (Sigma, Darmstadt, Germany), 0.2% Gentamycin and after achievement of confluence (3 days) the cells were able to be passaged or frozen at -80°C or liquid nitrogen.

### Collagen Staining of Cardiac Sections

Twenty one days after ischemia/reperfusion hearts were taken, embedded in paraffin and sections of these hearts were prepared. Scar formation was analyzed by staining of these sections with Gomori-, Bouin’s– (Sigma, Darmstadt, Germany) and hematoxylin solution (Dr. K. Hollborn & Söhne, Leibzig, Germany). Images were captured by Binocular Microscope (Nikon SMZ25), evaluated by Zen2 blue edition Software (Zeiss) and the ratio of the infarct size to the total of the left ventricle was determined.

To determine the amount of interstitial collagen, cardiac sections were stained by Picrosirius red staining (Morphisto, Frankfurt am Main, Germany) and interstitial collagen was measured in percent by area fraction. Additionally, Celestine-blue-solution (Sigma, Darmstadt, Germany) was used to stain the nuclei.

### Immunohistochemistry of Cardiac Sections

Twenty four hours after ischemia/reperfusion hearts were taken and paraffin sections of these hearts were stained either with Hematoxylin/Eosin (HE) solution (Sigma, Darmstadt Germany) or immune cell specific with a Streptavidinbiotin-immunoperoxidase method (Dako, Darmstadt, Germany). By HE-staining the total number of cells migrated into the infarcted area of the heart was counted per visual field and data are shown per mm^2^. For immune cell specific analysis paraffin-embedded heart sections were stained with an anti-Ly6G antibody for neutrophil staining (BD Pharmingen, Heidelberg, Germany) and an anti-Mac3 antibody for monocyte staining (BD Pharmingen, Heidelberg, Germany). From 12 areas of the infarct border zone either Mac3- or Ly6G-positive cells were counted and the data are shown per mm^2^.

For the analysis of α-SMA positive cells in the infarct area 21 days after ischemia/reperfusion, α-smooth muscle actin (α-SMA) staining of paraffin sections was performed using anti-α-SMA antibody (Abcam, Cambridge, United Kingdom), anti-rabbit horseradish peroxidase as second antibody (Santa Cruz, Frankfurt am Main, Germany) and Diaminobenzidine (DAB) reagent (DAKO, Darmstadt, Germany) as chromogen.

### Analysis of Cleaved Caspase-3 Activation

For the analysis of cleaved caspase-3 positive cells in cardiac sections 24 h after ischemia/reperfusion the streptavidinbiotin-immunoperoxidase method was performed again by staining with Cleaved Caspase-3 antibody (Cell Signaling, Frankfurt am Main, Germany). Caspase-3 positive cells were counted in the infarct border zone and data are presented per mm^2^.

To analyze caspase-3 activation *in vitro*, HL-1 cells were cultivated in Claycomb Medium supplemented with 10% fetal calf serum, 100 U/ml penicillin/streptomycin, 0.1 mM norepinephrine and 2 mM L-glutamine in T75 flasks coated with 0.02% gelatin and 0.5% fibronectin at 37°C with 5% CO_2_ in the atmosphere. Media was changed every 2 days and confluent flasks were trypsinized and split in a ratio of 1:3 for further subcultivation or subjected to the experimental procedures. For simulation of ischemia/reperfusion *in vitro*, HL-1 cells were subjected to 2 h ischemia and 5 h reperfusion with or without 1μM FIPI. Briefly, HL-1 cells were plated on 6-well plates (600,000 cells per well) and let adhere for 24 h. In order to simulate ischemia, cells were washed once and challenged with N_2_-pregassed acidified Krebs-Henseleit buffer (125 mM NaCl, 8 mM KCl, 1.2 mM KH_2_PO_4_, 1.25 mM MgSO_4_, 1.2 mM CaCl_2_, 6.25 mM NaHCO^3^, 20 mM HEPES, 5 mM Na-Lactate, pH 6.6) and 0.5% O_2_, 5% CO_2_, and 94.5% N_2_ at 37°C for 2 h in the humidified incubator of a hypoxic work chamber (Xvivo System, Biospherix, Parish, NY, United States). Reperfusion was mimicked by changing the medium to reperfusion buffer (O_2_-pregassed Krebs-Henseleit buffer: 110 mM NaCl, 4.7 mM KCL, 1.2 mM KH_2_PO_4_,1.25 mM MgSO_4_, 1.2 mM CaCl^2^, 25 mM NaHCO^3^, 20 mM HEPES 15 mM glucose, pH 7.4) and subjecting the cells to 21% O_2_ and 5% CO_2_ at 37°C for 5 h. As a time-matched non-I/R control, HL-1 cells were incubated in ungassed reperfusion buffer under non-ischemic conditions (21% O_2_, 5% CO_2_, 37°C) in parallel for 7 h. FIPI was added to respective cells immediately after the ischemic period during reperfusion. After simulation of ischemia/reperfusion cells were lysed and the determination of the expression level of caspase-3 was performed via Western blot analysis with anti-Cleaved-Caspase-3 (Cell Signaling, Frankfurt am Main, Germany) and Anti-GAPDH antibody (Cell Signaling, Frankfurt am Main, Germany) as control.

#### IL-1β/TNF-α Enzyme-Linked Immunosorbent Assay (ELISA)

For quantification of IL-1β in plasma 24 h after MI, heparinized blood was centrifuged 10 min for 650 g. The plasma was taken and the cytokine amount was measured by Enzyme-Linked Immunosorbent Assay following the manufacturer’s protocol (DuoSet Mouse IL-1β/IL-1F2 ELISA, R&D Systems, Minneapolis, MN, United States).

To investigate the release of TNF-α, PLD activity in MEFs was inhibited by treatment with 1 μM FIPI (5-Fluoro-2-indolyl des-chlorohalopemide, Darmstadt, Germany) for 30 min. After the stimulation of MEFs with 1 μg/ml LPS (lipopolysaccharide, Sigma, Darmstadt, Germany) for different time periods as indicated, TNF-α amount was measured in the cell culture supernatant by sandwich–ELISA, following the manufacturer’s protocol (DuoSet Mouse TNF-α ELISA; R&D Systems, Minneapolis, MN, United States).

### FACS Analysis of Platelet-Immune Cell Aggregate Formation

Zero, Twenty four, and seventy two hours after MI the formation of platelet-immune cell aggregates was measured via flow-cytometry. Heparinized blood was washed twice with Tyrode’s Buffer, centrifuged 5 min for 650 g and only the hematocrit was used for measurements. Samples were incubated with either PE- or APC-conjugated antibodies for platelets (GPIb-PE, Emfret, Eibelstadt, Germany), neutrophiles (Ly6G-APC, Biolegend, Koblenz, Germany) and leucocytes (CD45-APC, BD Bioscience, Heidelberg, Germany) labeling. Data show the MFI (mean of fluorescence) of either the leucocyte or neutrophil-signal of double-positive cells.

### Quantitative Real-Time PCR

For the analysis of endogenously expressed levels of TNF-α, IL-6, Bax, Bcl-xl and Bcl-2 only isolated total RNA of the left ventricle of the heart 24 h after ischemia of wildtypic, FIPI-treated and *Pld1^-/-^/Pld2^-/-^* mice was used. RNA-Isolation was performed by ReliaPrep RNA Tissue Miniprep System (Promega, Mannheim, Germany) following the manufacturer’s protocol.

In addition, the TNF-α levels of LPS-stimulated MEFs from *Pld1^+/+^*- or *Pld1^-/-^* mice were measured by qRT-PCR. Therefor MEFs were stimulated with 1 μg/ml LPS for 12 h and RNA was isolated by following the manufacturer’s protocol of the RNeasy Mini Kit (Qiagen, Hilden, Germany).

Quantitative Real-time PCR was performed by using Fast Sybr Green Master Mix (Life Technologies, Carlsbad, CA, United States) following the manufacturer’s protocol. The expression level of the target was normalized to glyceraldehyde-3-phosphate dehydrogenase (GAPDH) RNA expression levels as a control. After reverse transcription, quantitative PCR amplification was performed using the following oligonucleotide primers: GAPDH for ′GGTGAAGGCGGTGTGAACG′; GAPDH rev ′CTCGCTCCTGGAAGATGGTG′; IL–6 for ′ACTCGGCAAACCTAGTGCGTTATG′; IL–6 rev ′ACATTCCAAGAAACCATCTGGCTAG′; BAX for ′TGAAGACAGGGGCCTTTTTG′; BAX rev ′AATTCGCCGGAGACACTCG′; Bcl–xl for ′GACAAGGAGATGCAGGTATTGG′; Bcl–xl rev ′TCCCGTAGAGACCACAAAAGT′; Bcl–2 for ′ATGTGTGTGGAGAGCGTCAA′cl–2 rev ′CATGCTGGGGCCATATAGTT′; TNF–α for ′GCCCCCATCTGACCCC-TTT′; TNF–α rev ′GGGGCTGGCTCTGTGAGGAA′.

### Statistical Analysis

All experiments were performed at least three times with n defined as individual animal. Data are presented as means ± SEM as indicated. Statistical analysis was performed using the two-tailed Student’s *t*-test, where at a *P* < 0.05 was set as significant. For all figures ^∗^*P* < 0.05, ^∗∗^*P* < 0.01, and ^∗∗∗^*P* < 0.001.

## Results

### Unaltered Infarct Size and Cardiac Function After FIPI Treatment of Mice 24 h Post MI

**FIGURE 1:**
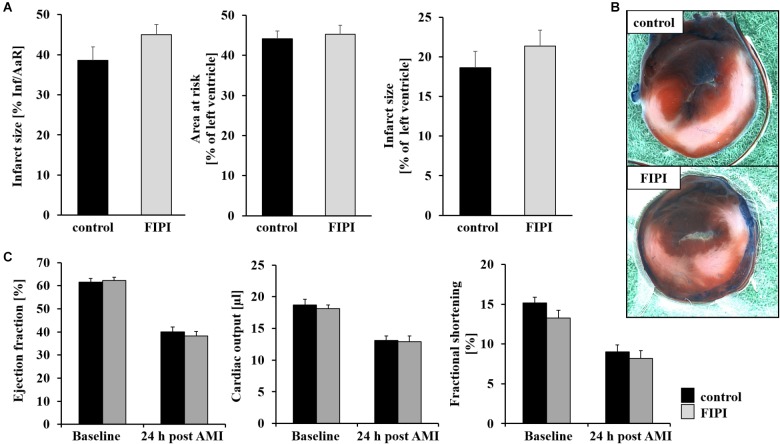
FIPI has no influence on infarct size or left ventricular function 24 h post MI in mouse hearts. **(A)** Quantitative analysis of infarct size as the percentage of area at risk (% Inf/AaR), of the area at risk as percentage of the left ventricle and of the infarct size as percentage of the left ventricle showed no differences in comparison of the control and FIPI-treated group in TTC-stained left ventricles 24 h after I/R (control *n* = 12, FIPI *n* = 13). **(B)** Representative pictures. Blue = healthy tissue, red = the area at risk (AAR), white = infarcted area (INF). **(C)** Echocardiographic analysis of ejection fraction (baseline vs. 24 h after I/R), cardiac output (baseline vs. 24 h after I/R) and fractional shortening (baseline vs. 24 h after I/R) of C57BL6/J mice treated with FIPI (3 mg/kg bodyweight in 4% DMSO/PBS) vs. controls showed no differences (control *n* = 20, FIPI *n* = 23).

It has been shown recently that PLD1 plays an important role in TNF-α mediated inflammation and scar formation after MI in mice (Schonberger et al., 2014). To investigate the impact of the enzymatic activity of PLD in the processes of I/R injury, we treated C57BL/6 mice with FIPI, a small reversible PLD inhibitor known to inhibit the enzymatic activity of both PLD1 and PLD2 (Su et al., 2009), and analyzed the mice in a model of myocardial I/R (Figure 1). The specificity and effectiveness for the inhibition of PLD by FIPI was already confirmed in different studies (Su et al., 2009; Dall’Armi et al., 2010; Stegner et al., 2013). After ligation of the LAD for 60 min, reperfusion was allowed for 24 h and myocardial damage was assessed by 2,3,5-triphenyltetrazolium chloride staining to differentiate between metabolically active and inactive tissue. The ischemic area (*area of risk*) and the infarcted area (*infarct size*) were determined and the ratios of the different areas were quantified digitally by video planimetry. No differences were observed between FIPI-treated and control mice (Figures 1A,B). Accordingly, cardiac function as determined by ejection fraction, cardiac output and fractional shortening were not different between both groups (Figure 1C).

### Enzymatic Activity of PLD Is Required for the Migration of Inflammatory Cells Into the Infarct Border Zone 24 h Post MI

**FIGURE 2:**
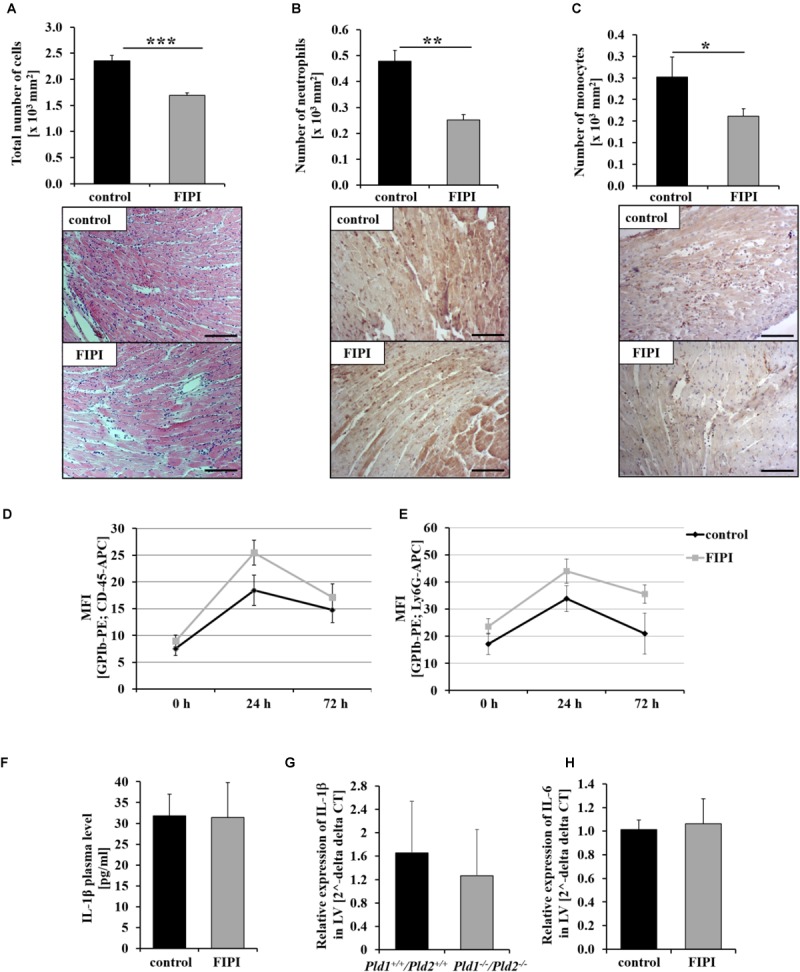
Pharmacological inhibition of PLD enzymatic activity reduces the inflammatory response 24 h post MI in mice. Twenty four hours post MI, cardiac sections of FIPI-treated mice (3 mg/kg bodyweight in 4% DMSO/PBS) vs. controls were stained with either hematoxylin/eosin **(A)** or immune cell specific antibodies for neutrophils **(B)** and macrophages **(C)** to analyze the migration of inflammatory cells into the infarct border zone (n = 5). ^∗^*P* < 0.05, ^∗∗^*P* < 0.01, ^∗∗∗^*P* < 0.001. **(D,E)** Flow cytometric analysis of platelet-immune cell-aggregate formation 0, 24, and 72 h post MI in FIPI-treated mice vs. controls; Platelet – leukocyte-aggregate formation left, platelet – neutrophile – aggregate formation right (*n* = 5–21). **(F)** Quantitative analysis of IL-1β in plasma of FIPI-treated mice vs. control 24 h post MI (control n = 14; FIPI n = 16). **(G)** Analysis of IL-1β expression in the left ventricle of *Pld1^+/+^/Pld2^+/+^* vs. *Pld1^-/-^/Pld2^-/-^* mice 24 h post MI using quantitative RT-PCR (*Pld1^+/+^/Pld2^+/+^ n* = 3, *Pld1^-/-^/Pld2^-/-^ n* = 4). **(H)** Quantitative analysis of IL-6 expression in the left ventricle of FIPI-treated vs. control mice 24 h post MI (control *n* = 5; FIPI *n* = 6). Scale bar: 50 μm, magnification: 400×.

Next, we examined the migration of inflammatory cells into the infarct border zone 24 h after MI. The analysis of cardiac sections revealed significantly reduced migration of cells in FIPI-treated mice compared to controls as observed by Hematoxylin/Eosin-staining (Figure 2A). Alterations in leukocyte cell migration included neutrophils (Figure 2B) and monocytes (Figure 2C) as already observed in PLD1 deficient mice (Schonberger et al., 2014), where migration of these cells 24 h post MI was reduced as well. However, the formation of platelet-leukocyte conjugates (Figure 2D) and platelet-neutrophil conjugates (Figure 2E) was not altered in FIPI-treated compared to control mice suggesting that platelet-mediated effects on leukocytes are not responsible for altered leukocyte migration post MI. No differences in the formation of platelet-immune cell-aggregates was observed in PLD1/PLD2 double deficient mice as well (Supplementary Figure S1). Moreover, we detected comparable IL-1β levels in plasma of FIPI-treated mice compared to controls (Figure 2F). In line with this result, the expression of IL-6 in the left ventricle of mice 24 h post MI was unaltered (Figure 2H) suggesting that the enzymatic activity of PLD has no impact on acute phase cytokine expression and release post MI. However, the expression of IL-1β in the left ventricle of PLD1/PLD2 double deficient mice was not altered as well (Figure 2G).

### PLD Enzymatic Activity Does Not Affect Cell Survival 24 h Post MI

**FIGURE 3:**
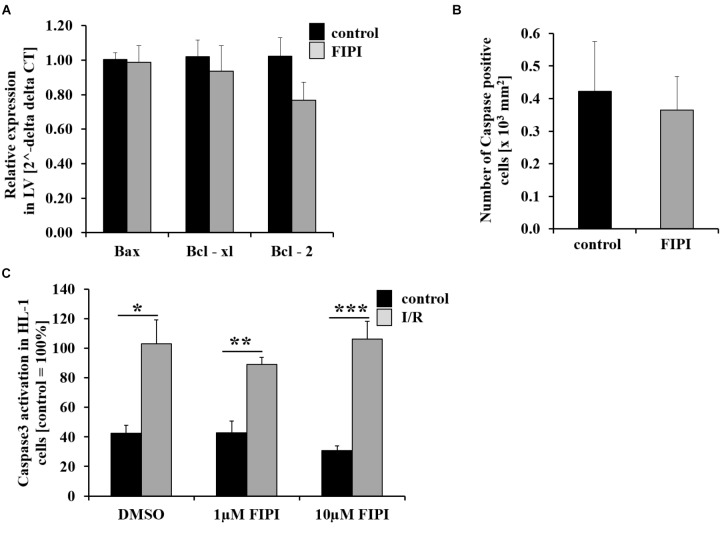
Treatment with FIPI shows no alterations in cell apoptosis after I/R. **(A)** Analysis of quantitative RT-PCR shows no differences in the expression of the cell apoptosis markers *Bax, Bcl-xl*, and *Bcl*-2 in the left ventricle (LV) of FIPI-treated mice (3 mg/kg bodyweight in 4% DMSO/PBS) vs. control mice 24 h post MI (control *n* = 5, FIPI *n* = 6). **(B)** Number of caspase-3 positive cells in the infarct border zone is not different between FIPI-treated vs. control mice 21 days post MI (control *n* = 4, FIPI *n* = 5) **(C)** After I/R *in vitro* the amount of activated caspase3 in HL-1 cells is not altered after FIPI-treatment (*n* = 4). ^∗^*P* < 0.05, ^∗∗^*P* < 0.01, ^∗∗∗^*P* < 0.001.

Apoptosis plays a role in the process of tissue damage after MI. To investigate whether pharmacological inhibition of PLD alters cell apoptosis after I/R, we determined the expression of different anti- and pro-apoptotic markers in the left ventricle of mice 24 h post MI using quantitative RT-PCR. As shown in Figure 3, the expression level of the apoptosis regulator Bcl-2-associated X protein (Bax) and the anti-apoptotic markers Bcl-xL and Bcl-2 were not altered in FIPI-treated mice compared to controls (Figure 3A). Accordingly, we detected no differences between FIPI-treated and control mice when we determined the number of caspase-3 positive cells in the infarct zone 24 h post MI (Figure 3B). We next performed another experiment to confirm the results from histology and analyzed caspase-3 positive cells *in vitro* using HL-1 cells that were subjected to 2 h ischemia and 5 h reperfusion with or without different doses (1 or 10 μM) of FIPI. FIPI was added to respective cells immediately after the ischemic period during reperfusion, cells were lysed and the expression level of caspase-3 was performed via Western blot analysis. A significant increase in the amount of caspase-3 was detected after I/R in all samples compared to controls without ischemia. However, there was no difference between DMSO controls and FIPI-treated cells with all concentrations tested in this assay (Figure 3C).

### FIPI Treatment Has No Impact on Scar Formation and Cardiac Function 21 Days Post MI

**FIGURE 4:**
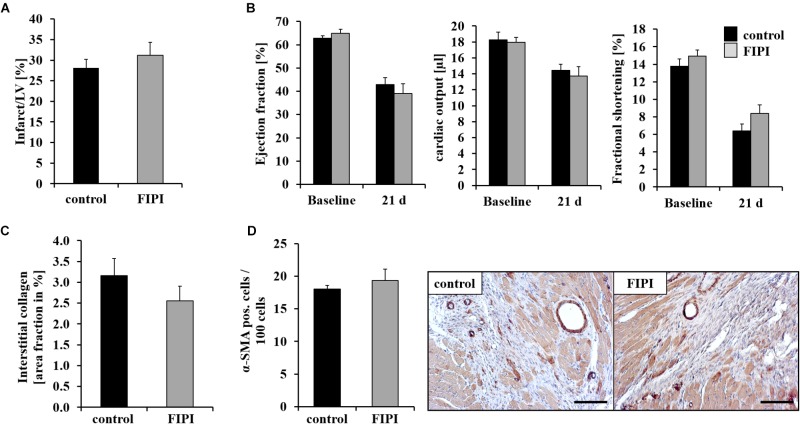
FIPI treatment has no influence on scar formation 21 d post MI. **(A)** No alterations in infarct size (*n* = 6) and **(B)** left ventricular function 21 days post MI in FIPI (3 mg/kg bodyweight in 4% DMSO/PBS) treated mice vs. controls (*n* = 14–18). **(C)** Sirius red staining of remote myocardium shows no differences in the amount of interstitial collagen between FIPI-treated mice vs. controls (control *n* = 9, FIPI *n* = 6) **(D)** Quantification of α-SMA positive myofibroblasts in the border zone of infarcted hearts 21 days post MI in FIPI-treated mice vs. control mice shows no alterations between the groups. Representative pictures are shown (control *n* = 9, FIPI *n* = 6). Scale bar: 50 μm, magnification: 400×.

To investigate the consequences of reduced migration of inflammatory cells into the infarct border zone 24 h post MI in FIPI-treated mice, we next analyzed cardiac damage and repair 21 days post MI. Determination of infarct size and cardiac function as measured by ejection fraction, cardiac output and fractional shortening was not different in FIPI-treated mice compared to controls (Figures 4A,B). Additional hemodynamic parameters 24 h and 21 days post MI were measured by echocardiography and are presented in Table 1 showing no differences between FIPI and DMSO treated mice.

**Table 1:** Hemodynamic measurements after MI.

*n* = 3–20	Heart rate (bpm)	Stroke volume (μl)	End-systolic volume (μl)	End-diastolic volume (μl)
**BASAL**				
C57BL/6J control	469.36 12.22	39.53 1.49	24.69 1.38	64.24 2.07
C57/Bl/6J + FIPI	478.72 10.96	37.994 1.14	23.36 1.43	61.36 2.08
**24 h POST MI**				
C57BL/6J control	474.87 10.27	27.58 1.28	42.35 2.36	69.96 1.95
C57/Bl/6J + FIPI	439.05 22.6	28.35 1.73	45.63 1.92	73.98 1.96
**21 DAYS POST MI**				
C57BL/6J control	514.25 7.95	28.11 1.46	39.33 3.42	67.44 1.79
C57/Bl/6J + FIPI	517.34 17.61	27.199 2.34	48.77 7.12	75.67 5.7

Fibroblasts are responsible for collagen deposition in the heart post MI. Therefore, we quantified interstitial fibrosis in non-infarcted regions. As shown in Figure 4C, FIPI treatment of mice did not alter the formation of interstitial collagen as determined by Sirius red staining of the remote myocardium (Figure 4C). Moreover, we immunostained cardiac myofibroblasts with α-SMA 21 days post MI. Treatment of mice with FIPI did not induce alterations in the number of α-SMA positive cells (Figure 4D).

### Enzymatic Activity of PLD Is Not Responsible for PLD Mediated TNF-α Regulation Upon Inflammation

**FIGURE 5:**
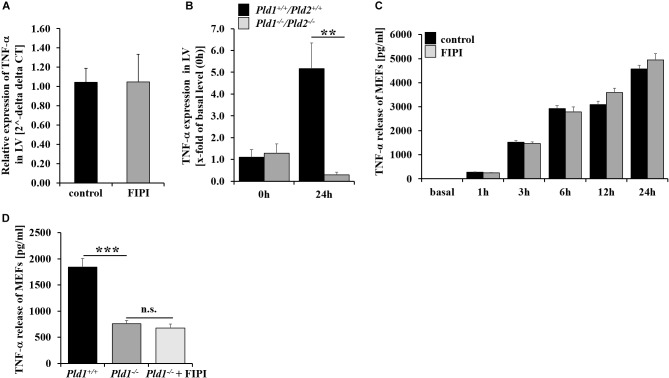
TNF-α signaling is independent of the enzymatic activity of PLD. **(A)** TNF-α expression in the left ventricle of infarcted hearts shows no alterations in FIPI-treated (3 mg/kg bodyweight in 4% DMSO/PBS) vs. control mice 24 h post MI (control *n* = 5, FIPI *n* = 6). **(B)** TNF-α expression in the left ventricle of *Pld1^+/+^/Pld2^+/+^* vs. *Pld1^-/-^/Pld2^-/-^* mice 24 h post MI was significantly decreased in mutant mice (*Pld1^+/+^/Pld2^+/+^ n* = 3/4; *Pld1^-/-^/Pld2^-/-^ n* = 3/5). **(C)** *Pld1^+/+^*-MEFs were treated with FIPI (1 μM in DMSO/PBS, 30 min prior LPS treatment), stimulated with LPS (1 μg/ml) for different time periods as indicated and the TNF-α release was measured via ELISA. No differences in TNF-α release were observed in FIPI-treated MEFs compared to untreated controls (*n* = 5). **(D)** *Pld1^-/-^-* MEFs were treated with FIPI (1 μM in DMSO/PBS) and stimulated with LPS (1 μg/ml) for 12 h. TNF-α release was measured by ELISA. TNF-α release of *Pld1^-/-^-MEFs* and *Pld1^-/-^-MEFs* treated with FIPI was decreased in comparison to *Pld1^+/+^-*MEFs (*n* = 5). LV = left ventricle. ^∗∗^*P* < 0.01 and ^∗∗∗^*P* < 0.001.

PLD1 is crucial for TNF-α mediated inflammation after MI and LPS-induced sepsis in mice (Schonberger et al., 2014; Urbahn et al., 2018). To investigate if enzymatic activity of PLD is important for TNF-α regulation, we determined TNF-α expression in the left ventricle of FIPI-treated mice. FIPI treatment of mice did not lead to alterations in TNF-α expression compared to control mice (Figure 5A). In contrast, TNF-α expression was significantly reduced in PLD1/PLD2 double deficient mice 24 h post MI suggesting that enzymatic activity of PLD is not responsible for PLD mediated TNF-α regulation upon inflammation (Figure 5B). Accordingly, the analysis of MEFs treated with FIPI showed no differences in TNF-α release after stimulation with LPS (Figure 5C). In contrast, LPS stimulation of PLD1 deficient MEFs resulted in significantly reduced TNF-α release (Figure 5D). However, FIPI treatment of PLD1 deficient MEFs did not further decrease the amount of TNF-α in the supernatant of LPS stimulated MEFs (Figure 5D).

## Discussion

The present study revealed that the enzymatic activity of PLD is not required for PLD mediated TNF-α regulation upon inflammation and scar formation after I/R. Consequently, infarct size and cardiac function was not altered in FIPI-treated mice as observed for PLD1 deficient mice that display enhanced infarct size and declined ejection fraction and fractional shortening following myocardial ischemia (Schonberger et al., 2014).

Different studies in the past identified PLD1 as regulator of TNF-α expression and release. PLD1 is required for LPS-induced TNF-α expression and production in Raw 264.7 cells (Oh et al., 2015). In rheumatoid arthritis synovial fibroblasts (RASF), PLD enzymes facilitate IL-17 and TNF-α induced expression of pro-inflammatory genes (Friday and Fox, 2016). PLD activation in natural killer cells modulates TNF-α synthesis via phosphatidic acid. However, here we provide strong evidence that PLD1-induced regulation of TNF-α is independent of its enzymatic activity, because FIPI treatment does not alter TNF-α expression and release in mice and in MEFs. Interestingly, PLD1 mediates TNF-α regulation via MEK1 and ERK phosphorylation leading to reduced Egr1 expression after LPS-induced sepsis in PLD1 deficient mice (Urbahn et al., 2018).

Lipase-independent roles of PLD were described in the last years. Park et al. (2015) showed that PLD2 promotes degradation of hypoxia-inducible factor (HIF)-1a independent of lipase activity by regulating the stability of HIF-1a through the dynamic assembly of HIF-1a, prolyl hydroxylase (PHD)2 and von Hippel-Lindau (VHL) protein. Thus, PLD1-mediated TNF-α regulation might be the result of PLD1-induced formation and/or stabilization of a phosphorylation complex that allows the phosphorylation of MEK and/or ERK including the interaction of proteins via the pleckstrin homology domain of PLD. Another possible mechanism how PLD1 regulates TNF-α independent of its enzymatic activity might be the modulation of the kinase activity responsible for the phosphorylation of MEK and ERK as observed by Ahn et al. (2003) who demonstrated that PLD1 significantly increases c-Src kinase activity.

In the processes of MI, genetic ablation of PLD1 resulted in defective migration of inflammatory cells into the infarct border zone, reduced TNFα and TGF-β secretion and smooth muscle α-actin expression of cardiac fibroblast leading to altered myofibroblast differentiation and interstitial collagen deposition in PLD1 deficient mice (Schonberger et al., 2014). As a result, infarct size and cardiac function were impaired in PLD1 deficient mice 28 days after MI indicating that PLD1 is crucial for inflammation and TGF-β mediated collagen scar formation to augment LV function after ischemia and reperfusion (Schonberger et al., 2014). However, blocking the enzymatic activity of PLD with FIPI does not alter scar formation and LV function but induces a defective inflammatory response after ischemia/reperfusion. Thus, the phenotype observed in PLD1 deficient mice is related to enzymatic and non-enzymatic properties of PLD1 after myocardial ischemia and reperfusion injury.

In contrast to TNF-α regulation, the migration of inflammatory cells depends on its lipase activity, because we detected a reduced number of neutrophils and monocytes into the infarct border zone of FIPI-treated mice as already observed in PLD1 deficient mice 24 h post MI (Figure 2; Schonberger et al., 2014). Surprisingly, Kim et al. (2006) provided evidence for PLD1 mediated regulation of cell migration to be independent of the lipase activity. The authors used small interfering RNA to silence PLD1 in HeLa cells. In contrast to recently published data showing no influence of PLD1 in cytoskeletal reorganization of platelets (Klier et al., 2017), Kim et al. (2006) found altered stress fiber formation and number of focal adhesions in these cells when PLD1 was silenced. Moreover, decreased migratory potential could be restored by adding wildtype or lipase-inactive PLD1 into knockdown cells. Here we showed that pharmacological inhibition of PLD reduced migration of inflammatory cells as observed for PLD1 deficient mice suggesting that PLD mediated adhesion and migration of these cells depends on the enzymatic activity of PLD (Schonberger et al., 2014; Urbahn et al., 2018). However, there might be differences in diverse cells. Moreover, cell migration was analyzed in mice after I/R injury and LPS-induced sepsis showing reduced migration of inflammatory cells in both mouse models. In contrast, Kim et al. (2006) used tumor cells (HeLa cells) and analyzed cell migration *in vitro*. These differences in cell types and in the experimental set-up might all account for different results.

TNF-α contributes to I/R injury and post MI remodeling but also to cardioprotection by ischemic conditioning suggesting an ambivalent role of TNF-α (Kleinbongard et al., 2010). In line with the important role of TNF-α after MI, we did not find altered scar formation or cardiac function in FIPI-treated mice. This suggests that decreased TNF-α plasma levels are – at least in part – responsible for increased infarct size and declined cardiac function in PLD1 deficient mice, that displays significantly reduced TNF-α plasma levels 24 h after MI (Schonberger et al., 2014). However, as FIPI inhibits the enzymatic activity of both, PLD1 and PLD2, it is tempting to speculate if PLD2 plays any role in scar formation and cardiac function after I/R injury.

Reduced migration of inflammatory cells as observed in PLD1 deficient and in FIPI-treated mice might be due to PLD-induced integrin activation. As Stegner and colleagues showed that FIPI treatment of mice leads to protection from occlusive thrombus formation as observed for PLD1 deficient mice caused by a GPIb-depending integrin defect, modulation of integrin activation might be the result of PLD lipase activity (Elvers et al., 2010; Stegner et al., 2013). In line with these results, Powner and colleagues already found in 2007 that stable adhesion and migration of neutrophils requires PLD-mediated activation of integrins (Powner et al., 2007).

Stegner et al. (2013) claimed that pharmacological inhibition of PLD might be a safe therapeutic strategy to prevent arterial thrombosis. If FIPI treatment would induce the same phenotype after MI as observed in PLD1 deficient mice including increased myocardial damage and declined cardiac function, the therapeutic potential of FIPI would be questioning. However, our data provide evidence that blocking of the enzymatic activity of PLD has no translational potential in patients with MI because inhibition of PLD enzymatic activity does lead to neither altered cardiac function nor to altered infarct size, at least in mice. This suggests that patients with MI will not have any benefit from a FIPI-based therapy with regard to improved cardiac function.

## Author Contributions

MK, M-AU, and DB performed the experiments. SG did I/R and TTC-staining. MK and ME discussed the data and wrote the manuscript. All authors reviewed and contributed to the manuscript.

## Conflict of Interest Statement

The authors declare that the research was conducted in the absence of any commercial or financial relationships that could be construed as a potential conflict of interest.
