# Supplementary material for: Enzymatic Activity Is Not Required for Phospholipase D Mediated TNF-α Regulation and Myocardial Healing
Source: Front Physiol. 2018 Nov 29;9:1698. doi: 10.3389/fphys.2018.01698 (PMC6281985; doi:10.3389/fphys.2018.01698)
Supplement: Supplementary file 1 [file Presentation_2.pdf]

Supplemental Data

Enzymatic activity is not required for phospholipase D mediated TNF-α regulation and myocardial healing

M. Klier[1], S. Goressen[2], M. Urbahn[1], D. Barbosa[3], M. Ouwens[3], M. Elvers[1]

Supplemental Figure Legends

S1: Lacking of PLD does not alter the formation of platelet-immune cell-aggregates.

(A) Flow cytometric analysis of platelet-immune cell-aggregate formation 0, 24 und 72 hours post AMI in *Pld1*$^{-/-}$/*Pld2*$^{-/-}$ mice vs. *wt*; Platelet – leukocyte-aggregate formation left, platelet – neutrophile – aggregate formation right (*Pld1*$^{+/+}$/*Pld2*$^{+/+}$ n = 6; *Pld1*$^{-/-}$/*Pld2*$^{-/-}$ n = 9).

A

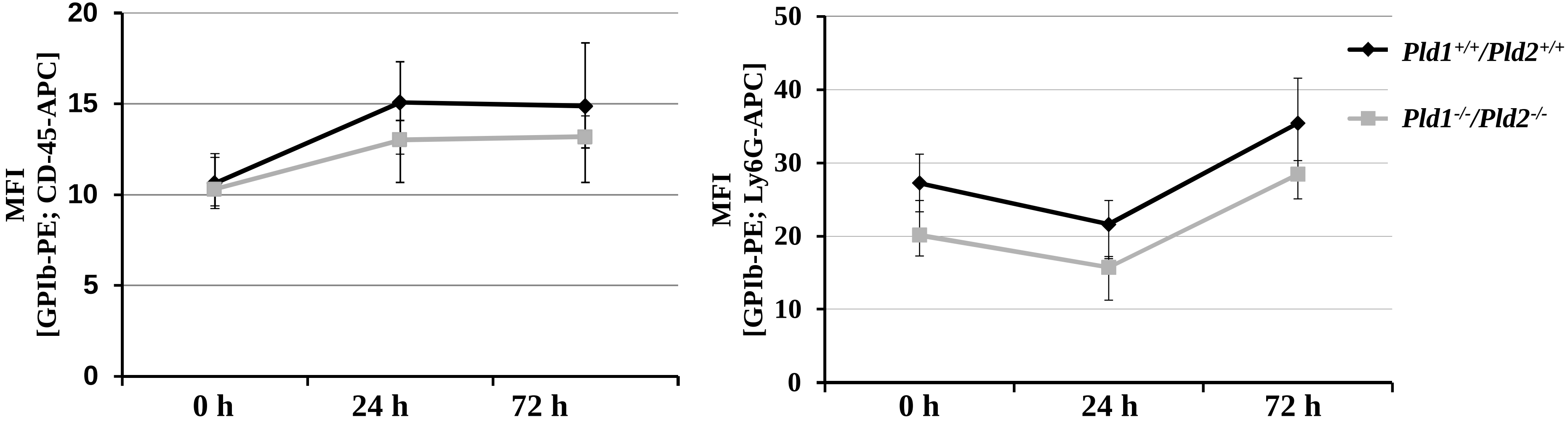
20
15
10
5
0
MFI
[GPIb-PE; CD-45-APC]
0 h
24 h
72 h
50
40
30
20
10
0
MFI
[GPIb-PE; Ly6G-APC]
Pld1+/+/Pld2+/+
Pld1-/-/Pld2-/-

Suppl.-Fig.1
